# Pityriasis Lichenoides et Varioliformis Acuta: A Case of Iatrogenic Cushing’s Syndrome Induced by Topical Corticosteroids

**DOI:** 10.7759/cureus.108741

**Published:** 2026-05-12

**Authors:** Cristina Palacio, Sara Saldarriaga Santamaría, Catalina Olarte, Victoria Davila

**Affiliations:** 1 General Medicine, Universidad CES, Medellin, COL; 2 Dermatology, Universidad CES, Medellin, COL

**Keywords:** corticosteroid, cushing’s syndrome, hypercortisolism, pediatric, pityriasis lichenoides

## Abstract

The use of topical corticosteroids plays a central role in the management of inflammatory skin disorders in dermatology, owing to their cost-effectiveness and early symptom resolution. However, inappropriate or prolonged use, particularly of high-potency agents, may lead to local and systemic adverse effects, including the suppression of the hypothalamic-pituitary-adrenal axis. Although systemic complications are likely underreported in the literature, the pediatric population is particularly prone to experiencing them. We present the case of an eight-year-old boy with skin lesions compatible with pityriasis lichenoides et varioliformis acuta (PLEVA) who developed clinical features of iatrogenic Cushing’s syndrome following 10 days of high-potency topical corticosteroids.

## Introduction

Over time, topical corticosteroids have become a cornerstone in the treatment of inflammatory disorders in dermatology. When used in targeted therapeutic regimens, they help reduce most inflammatory lesions, standing out for their cost-effectiveness in acute conditions [1].

However, the use of these medications, especially in different potency grades and for prolonged periods, has been associated with side effects such as acne, telangiectasias, rosacea, striae, and atrophy, particularly in sensitive areas like the face and genitals. Similarly, systemic manifestations such as adrenal axis suppression and associated hypercortisolism can occur [1,2].

It is particularly important to exercise caution when using corticosteroids in the pediatric population, as their morphological skin characteristics allow for greater absorption, making them more prone to adverse effects. While local reactions are the most common presentation, systemic effects are less frequent but still a concern [2,3].

Pityriasis lichenoides (PL) is a spectrum of inflammatory skin diseases encompassing two main subtypes: pityriasis lichenoides et varioliformis acuta (PLEVA) and pityriasis lichenoides chronica (PLC), each with distinct clinical presentations and courses. Its underlying etiology remains a subject of ongoing debate, involving potential inflammatory responses to infectious agents or T-cell lymphoproliferative disorders, and no definitive consensus has been reached to date [4,5].

Its precise incidence remains poorly established in the literature. A case series conducted at a university hospital in Serbia identified 242 patients diagnosed with PL, of whom 135 were children, and 107 were adults, suggesting a higher prevalence in the pediatric population. Notably, PL predominantly affects males among children, with a statistically significant higher incidence compared to female patients (p < 0.01). Regarding potential triggering factors, a preceding viral infection was identified in 15% of cases; however, no association was established between immunizations and disease onset or subsequent relapses [6].

This case report describes a pediatric patient with pruritic skin lesions suggestive of PLEVA who, after treatment with a high-potency steroid (clobetasol) for 10 days, developed clinical signs of iatrogenic Cushing's syndrome.

This article was previously posted to the Authorea preprint service on January 24, 2025.

## Case presentation

An eight-year-old male patient, with no significant past medical history, presented to pediatric dermatology with a one-month history of pruritic skin lesions. Physical examination revealed multiple erythematous macules and papules, some with necrotic crusts, distributed symmetrically on the chest and abdomen.

**Figure 1:**
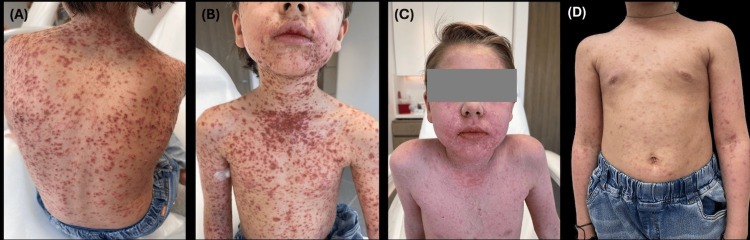
Multiple symmetrical oval and round lesions, reddish-brown in color, including macules, papules, necrotic areas, and crusts on the face, trunk, and extremities at initial presentation (A, B). Clinical signs of iatrogenic Cushing's syndrome, including moon facies, alongside the evolution of the skin lesions following 10 days of high-potency corticosteroid therapy (C, D).

With a presumptive diagnosis of PLEVA, a skin biopsy was performed, and the patient was started on erythromycin at 40 mg/kg/day. However, two weeks later, the lesions had spread to the face, extremities, genitals, palms, and soles, accompanied by vesicles and papules with hemorrhagic crusts (Figures 1A, 1B).

Histopathology confirmed the suspected diagnosis, and given the severity and extent of the disease, treatment was escalated to methotrexate (0.4 mg/kg weekly) with folic acid and clobetasol propionate cream 0.05% nightly over 30% of the body surface area without occlusion, resulting in notable improvement within two weeks.

Ten days after starting nightly clobetasol application, the patient's parents noticed progressive facial swelling. Follow-up examination revealed moon facies, facial plethora, and a 1.5 kg weight gain (Figures 1B, 1C). Suspecting iatrogenic Cushing's syndrome secondary to high-potency corticosteroid use, a 24-hour urine free cortisol test was performed, with levels above the baseline values for this age, therefore confirming Cushing's syndrome. The corticosteroid therapy was tapered, and methotrexate was continued along with folic acid. The patient showed satisfactory improvement and clinical recovery at three-month follow-up (Figure 1D).

## Discussion

Topical corticosteroids are widely used in pediatric dermatology due to their anti-inflammatory action, cost-effectiveness, and safety. These agents are easily absorbed into the dermis, with absorption increasing significantly in inflamed skin [7,8].

Clobetasol propionate is among the most potent topical corticosteroids, classified as a super-high-potency agent (Class I) that is significantly more potent than hydrocortisone [8]. The use of 2 g/day of 0.05% clobetasol propionate can reduce morning cortisol levels within days, and weekly doses exceeding 100 g can lead to features of Cushing's syndrome and adrenal insufficiency. Several factors can increase the risk of systemic toxicity: corticosteroid potency, quantity and frequency of application, age, skin quality, presence of occlusion, and duration of use [9,10].

Several cases reported in the literature underscore the real risk of iatrogenic Cushing's syndrome (ICS) secondary to topical corticosteroid use across different patient populations, routes of application, and clinical contexts. While systemic administration remains the most commonly implicated route, cases have been documented with inhaled, intranasal, ophthalmic, and topical formulations, highlighting that no route is entirely exempt from systemic effects [11]. A particularly illustrative case involved a five-year-old male child with plaque psoriasis who developed cushingoid features after the application of 30 g/day of clobetasol propionate 0.05% for approximately one year [12]. At the other end of the clinical spectrum, even ophthalmic betamethasone eye drops have been reported to trigger exogenous Cushing's syndrome in infants [11]. Additionally, systemic adverse effects, including HPA (hypothalamic-pituitary-adrenal) axis suppression, have been documented following the application of clobetasol gel to the oral mucosa [13]. Finally, chronic application in intertriginous areas warrants particular caution, as the naturally occlusive nature of skin folds increases percutaneous absorption [14].

This case highlights the risk of ICS secondary to topical corticosteroid use in the pediatric population. While ICS due to systemic corticosteroids is more commonly seen in practice, its occurrence following topical therapy, especially within such a short timeframe, is rarely reported. Risk factors present in this case include an altered skin barrier, a higher body surface area relative to weight, and the use of a high-potency molecule [15].

Similarly to our patient, Marín-Hernández et al. [16] reported a case of a five-year-old male child with PLEVA where the clinical presentation and initial histopathological findings posed a significant diagnostic challenge, initially mimicking mycosis fungoides. While their case emphasizes the risk of diagnostic ambiguity and the importance of expert histological review, our case highlights a different but equally critical clinical challenge: the potential for severe iatrogenic complications from high-potency topical therapy. Both cases underscore that PLEVA in the pediatric population requires not only diagnostic precision but also a highly cautious therapeutic approach to avoid secondary morbidity.

In the context of our patient’s clinical course, the aforementioned factors related to the skin barrier and treatment intensity likely increased the patient's risk of systemic toxicity, leading to ICS. This case emphasizes the importance of careful selection of the potency, formulation, and duration of topical corticosteroids in pediatric patients, particularly when applied to extensive areas with compromised skin barriers. Early recognition of clinical signs is crucial to prevent severe complications, and these patients require close monitoring to minimize risks associated with therapy [17].

Similar cases have been reported in infants treated for scabies, where the use of high-potency topical steroids led to rapid weight gain and clinical features of Cushing's syndrome within months [18].

The unique microstructural and physiological characteristics of the pediatric skin barrier, which explain the increased percutaneous absorption and subsequent systemic toxicity observed in this patient, are summarized in Table 1 [19].

**Table 1:** Comparative Microstructure of Infant vs. Adult Skin Barrier Table created by the authors. Source: [19]

Morphological Variable	Pediatric Skin	Adult Skin
Stratum Corneum (SC)	30% thinner; higher water content and transepidermal water loss. Micro-relief lines are denser, implying a larger surface per projected area.	Thicker and more compact layer; less surface area per projected area.
Corneocyte and Keratinocyte Size	Significantly smaller corneocytes in all sites; smaller keratinocytes at the granular layer.	Larger, more mature cells.
Epidermal Thickness	Supra-papillary epidermis is 20% thinner than in adults on average.	Significantly thicker cellular layer.
Hair Structure Density	10 times more hair structures per unit skin surface area in newborns.	Significantly lower follicle density per cm².
Dermal Collagen and Elastic Fibers	Absence of thick bundles in the upper reticular dermis; gradual transition between papillary and reticular layers.	Thicker fibers in the reticular dermis compared to the papillary layer.
Dermal Papillae	Homogeneous size; one-to-one relationship with surface "island" structures.	Irregular shape and size; one "island" corresponds to several papillae.

## Conclusions

Topical corticosteroids remain a cornerstone of dermatological therapy; however, their potential for systemic adverse effects demands continuous clinical vigilance, particularly in the pediatric population. Children are inherently more susceptible to these complications due to their higher skin surface area-to-body weight ratio, making early recognition of signs and symptoms essential. Their use in this population necessitates judicious selection of potency, strict limitation of treatment duration, and careful consideration of the application site. The lowest effective potency formulation should be prescribed for the shortest course possible, with periodic reassessment to preclude unnecessary therapeutic prolongation. Application should be restricted to affected areas, with particular caution over anatomical sites of enhanced percutaneous absorption, including the face, groin, and intertriginous areas. In infants, diapers must be acknowledged as occlusive dressings, as they substantially increase transdermal drug delivery. Prolonged use warrants monitoring of growth velocity and HPA axis integrity, given the disproportionately elevated systemic absorption risk inherent to pediatric patients.

Patient and caregiver education is equally essential; clear instructions regarding proper application technique, dosage, and treatment duration should be provided, as unsupervised or excessive use has been associated with ICS and adrenal suppression, even with topical formulations.
